# Characterization of Hospital Admissions During Immune Checkpoint Inhibitor Therapy: Insights From the ICOG Study

**DOI:** 10.1002/cam4.70582

**Published:** 2025-01-25

**Authors:** Jonas Paul Wiegmann, Tabea Fröhlich, Nora Möhn, Laura Duzzi, Emily Narten, Johanna Aurich, Janin Thomas, Lea Grote‐Levi, Susann Mahjoub, Dominik Berliner, Thomas Wirth, Heiko Golpon, Benjamin‐Alexander Bollmann, Imke Von Wasilewski, Ralf Gutzmer, Florian H. Heidel, Thomas Skripuletz, Gernot Beutel, Philipp Ivanyi, ICOG‐CCCH (Immune Cooperative Oncology Group, Comprehensive Cancer Center Hannover)

**Affiliations:** ^1^ Department of Hematology, Hemostasis, Oncology and Stem Cell Transplantation Hannover Medical School Hannover Germany; ^2^ ICOG‐CCCH (Immune Cooperative Oncology Group, Comprehensive Cancer Center Hannover) Hannover Germany; ^3^ Department of Neurology Hannover Medical School Hannover Germany; ^4^ Department of Cardiology and Angiology Hannover Medical School Hannover Germany; ^5^ Department of Gastroenterology, Hepatology, Infectious Disease and Endocrinology Hannover Medical School Hannover Germany; ^6^ Department of Respiratory Medicine and Infectious Disease Hannover Medical School Hannover Germany; ^7^ Department of Dermatology, Allergology and Venerology Hannover Medical School Hannover Germany; ^8^ Department of Dermatology, Johannes Wesling Medical Centre Minden Germany; ^9^ Interdisziplinäre Arbeitsgruppe Nierenzellkarzinom IAG‐N Deutsche Krebsgesellschaft Berlin Germany

**Keywords:** hospital admission, immune checkpoint inhibitor therapy, immune‐related adverse events, immuntherapy

## Abstract

**Introduction:**

Immune checkpoint inhibitors (ICI) have improved the therapeutic arsenal in outpatient oncology care; however, data on necessity of hospitalizations associated with immune‐related adverse events (irAEs) are scarce. Here, we characterized hospitalizations of patients undergoing ICI, from the prospective cohort study of the immune cooperative oncology group (ICOG) Hannover.

**Methods:**

Between 12/2019 and 06/2022, 237 patients were included. Clinical data and characteristics of ICI were collected during a 6‐month observation period after the initiation of therapy. Descriptive statistics and Kaplan–Meier statistics were administered.

**Results:**

During the observation period, 30/237 patients were hospitalized (HA(+)). Most common underlying tumor entities were malignant melanoma (59.5%), renal cell carcinoma (13.1%), and nonsmall‐cell lung carcinoma (12.7%). HA(+) patients exhibited an increased rate of pulmonary and cerebral metastases. We observed a significantly higher hospitalization rate during dual ICI with Nivolumab and Ipilimumab (*p* = 0.001). The predominant irAEs for hospitalization were colitis (26.7%), followed by hypophysitis (13.3%), leading to a median hospitalization of 7 (1–34) days. Interdisciplinary consultations were frequent, especially to gastroenterology (46.7%) and neurology (26.7%). Although a trend toward a prolonged overall survival in the HA(+) subgroup was identified, no statistically significant differences were found.

**Discussion:**

The hospitalization rate of 12.6% is comparable to rates reported in previous studies. There was a disproportionate admission of patients with immune‐related colitis and hypophysitis compared to the prevalence described under ICI. We observed a high need for interdisciplinary consultations in line with the heterogeneity of immune‐mediated side effects. Compared to non‐hospitalized patients, there was no survival disadvantage in the HA(+) cohort.

**Conclusion:**

With a relatively low hospitalization rate, short length of stay, and good clinical outcome, our data support the outpatient nature of ICI. The findings underscore the importance of interdisciplinary collaboration and vigilant monitoring of irAEs to ensure timely recognition and management.

## 1 Introduction

The advent of immune checkpoint inhibitors (ICI) has revolutionized the landscape of cancer therapy and established a new pillar of antitumor therapies [1, 2, 3]. Harnessing the power of the immune system to combat cancer, ICI have demonstrated remarkable efficacy in numerous clinical trials and real‐world settings [4, 5, 6, 7, 8, 9]. Key checkpoint molecules such as programmed cell death protein 1 (PD‐1), programmed death‐ligand 1 (PD‐L1), and cytotoxic T‐lymphocyte‐associated antigen 4 (CTLA‐4) have become focal points in the quest to unleash potent antitumor immune responses [10, 11]. ICI are used as monotherapies, dual checkpoint blockade, as well as in combination therapies with targeted tyrosine kinase inhibitors (TKIs) or chemotherapies [12, 13].

Due to the mechanism of ICI, immune‐related adverse events (irAEs) may occur during therapy. In addition to the most frequently described irAEs regarding the skin (rash, exanthema), the gastrointestinal tract (diarrhea, colitis), endocrine glands (hypo‐ or hyperthyreoiditis, hypophysitis), and liver (elevation of transaminases), the spectrum extends to rarer side effects such as pneumonitis, cardiomyopathy as well as neurological irAEs [14, 15, 16, 17, 18]. It is of fundamental importance to recognize relevant AEs and react promptly via dose delays, reduction, termination of ICI or even addition of immunosuppressive therapy [19].

In some cases, irAEs lead to the need for hospital treatment. The outpatient feasibility is considered a major advantage of ICI. For this reason, there is a need to evaluate the frequency, intensity and clinical outcome of inpatient treatment under ICI therapy. To date, there are few studies on this issue that show a hospitalization rate around 10% with an overall good clinical outcome [20, 21, 22, 23].

In this context, our study aimed to characterize hospital admissions of patients undergoing ICI therapy in a large tertiary center of interdisciplinary ICI patients, shedding light on the clinical features, management approaches, and outcomes of irAE‐related admissions.

## 2 Materials and Methods

### 2.1 Database—ICOG Study

Since December 2019, a prospective interdisciplinary cohort study has been running at the Hannover Medical School. The Departments of Hematology and Oncology, of Dermatology, of Neurology, of Respiratory Medicine, and of Gastroenterology at Hannover Medical School collaborated to form the immune cooperative oncology group (ICOG) of the Claudia von Schilling Comprehensive Cancer Center, which is responsible for all patients included in this study.

After receiving written informed consent, oncological patients from the aforementioned departments who had been elected for ICI therapy were prospectively included in this analysis. Patients with various cancer entities who were naive to ICI were included. Subgroup analyses were performed post hoc. ICI had to be administered at least once. ICI treatment was done according to Summary of Product Characteristic. Staging procedures were done according to local standard, evaluation was done according to radiological or clinical judgment. Demographic parameters, oncological or concomitant conditions, and clinical outcome were among the descriptive data of the study cohort.

Data were assessed according to the ICOG study protocol, each patient was examined at seven priorly specified time points: (i) before ICI therapy (baseline); (ii–vi) 3–4 weekly visits (V1, V2, …) after baseline; and (vii) 6 months after baseline (End of Study Visit [EOS]). Patients underwent a thorough clinical neurological assessment both at baseline and during EOS (Figure S1). Plasma‐ and serum‐containing blood samples were taken at baseline and at each subsequent visit, as well as EOS. After EOS patients had frequent follow‐ups regarding hospitalization, progression, and death. Based on the Common Criteria for Adverse Events (CTCAE) version 6.0, occurring irAEs were categorized into severity grades ranging from 1 (mild) to 5 (death because of adverse events). Hospital admission was checked during visits, EOS, and at the time of evaluation.

In accordance with the Declaration of Helsinki, the study protocol was authorized by the Ethics Committee at Hannover Medical School (no. 8685_BO_K2019).

### 2.2 Statistical Analysis

Descriptive and inferential statistics were used to analyze the data. For categorical data, absolute frequencies, percentages, and, if relevant, ranges were noted. *P* values were determined using Pearson's chi‐squared test, Mann–Whitney *U* test, and Student's *t*‐test as appropriate. Overall survival (OS) was defined as the period from the palliative medical treatment with ICI to the last visit or death. Progression‐free survival (PFS) was stated as the time from the palliative systematic treatment with ICI to the patient's demise or progression, based on radiological or clinical judgment. To calculate PFS and OS, Kaplan–Meier analysis, and log‐rank test were used. Cox‐proportional hazard regression analysis, both univariate and multivariate, were used to assess the significance of the variables. A multivariate regression analysis was performed on variables that showed a *p* < 0.2 in the univariate study. Confidence intervals (CIs) and hazard ratios (HRs) were calculated. *p* < 0.05 was the alpha threshold for significance testing. SPSS v29 (IBM Corp., Armonk, USA) was used for statistical analysis.

Patients included in this cohort started their treatment between December 2019 and June 2022, and were followed‐up until November 2022. If patients were hospitalized during their ICI therapy, they were included in the hospital admission (HA(+)) cohort. If patients were not admitted to the hospital in that timespan, they were included in the HA(−) cohort. The best objective response (BOR) was determined by radiological assessment. The outcome of the inpatient stay was defined by subjective clinical improvement and objective reduction of toxicity (according to CTCAE parameters). Resolved was only registered at complete regression of AEs.

## 3 Results

Between December 2019 and November 2022, 247 patients were evaluated, 10 were excluded from the analysis as they did not receive ICI therapy. Two hundred and thirty‐seven patients were successfully included in the study, of which 30 (12.7%) were hospitalized during the observation period (Figure S2).

### 3.1 Patient, Tumor, and Treatment Characteristics

In the study cohort, no difference between the patients with hospital admission (HA(+)) and the HA(−) group were detected in terms of age and gender. Overall cohort's most underlying tumor disease was malignant melanoma (59.5%), followed by renal cell carcinoma (RCC, 13.1%), nonsmall‐cell lung cancer (NSCLC, 12.7%), and head and neck squamous cell carcinoma (HNSCC, 7.6%). The subgroups had no significant differences in TNM stages at first diagnosis (Table S1) or baseline of ICI therapy. In terms of metastasis, the HA(+) patients exhibited an increased frequency of pulmonary and cerebral metastases. Cardiovascular, pulmonary, and autoimmune comorbidities were not significantly increased among the two subgroups. However, the relative number of neurological preexisting conditions was increased in the hospitalized population (*p* = 0.023). Regarding nicotine abuse, alcohol abuse, and other prior medication, the subgroups showed no difference (Table 1, Table S1).

**TABLE 1:** Patient characteristics at initiation of ICI of the total ICOG cohort and subgroups with (HA(+)) and without hospital admission (HA(−)).

Parameter	ICOG cohort (*n* = 237)	HA (+) (*n* = 30)	HA (−) (*n* = 207)	*p*
Age, median (range), years	65 (22–87)	63 (48–85)	65 (22–87)	0.595
Sex, *n* (%)				
Male	155 (65.4%)	21 (70%)	134 (64.7%)	0.571
Female	82 (34.6%)	9 (30%)	73 (35.3%)	
Solid neoplasia, *n* (%)				
Malignant melanoma	141 (59.5%)	23 (76.7%)	118 (57.0%)	0.202
RCC	31 (13.1%)	4 (13.3%)	27 (13.0%)	
NSCLC	30 (12.7%)	2 (6.7%)	28 (13.5%)	
HNSCC	18 (7.6%)	1 (3.3%)	17 (8.2%)	
Other^a^	17 (7.2%)	0	17 (8.2%)	
T stage, *n* (%)				
T1–T2	50 (21.1%)	6 (20%)	44 (21.3%)	0.635
T3–T4	95 (40.1%)	9 (30%)	86 (41.5%)	
Tx	92 (38.8%)	15 (50%)	77 (37.2%)	
N stage, *n* (%)				
N0	37 (15.6%)	4 (13.3%)	33 (15.9%)	0.840
N+	124 (52.3%)	12 (40.0%)	112 (54.1%)	
Nx	76 (32.1%)	14 (46.7%)	62 (30.0%)	
M stage, *n* (%)				
M0	72 (30.4%)	6 (20.0%)	66 (31.9%)	0.148
M1	136 (57.4%)	21 (70.0%)	115 (55.6%)	
Mx	29 (12.2%)	3 (10.0%)	26 (12.6%)	
Metastasis localization, *n* (%)				
Pulmonary	70 (29.5%)	16 (53.3%)	54 (26.1%)	**0.002**
Hepatic	32 (13.5%)	5 (16.7%)	27 (13%)	0.587
Cerebral	40 (16.9%)	12 (40%)	28 (13.5%)	**< 0.001**
Bone	35 (14.8%)	5 (16.7%)	30 (14.5%)	0.754
Soft tissue	19 (8%)	0	19 (9.2%)	0.084
Others	59 (24.9%)	10 (33.3%)	49 (23.7%)	0.253
Comorbidities, *n* (%)				
Cardiovascular	139 (58.6%)	15 (50%)	124 (59.9%)	0.303
Pulmonal	27 (11.4%)	2 (6.7%)	25 (12.1%)	0.383
Autoimmune	11 (4.6%)	1 (3.3%)	10 (4.8%)	0.716
Neurological	4 (1.7%)	2 (6.7%)	2 (1%)	**0.023**
Others	9 (3.8%)	1 (3.3%)	8 (3.9%)	0.887
Comedication 12 wks prior ICI, *n* (%)				
Steroids	5 (2.1%)	1 (3.3%)	4 (1.9%)	0.618
Antibiotics	2 (0.8%)	1 (3.3%)	1 (0.5%)	0.111
PPI	49 (20.7%)	8 (26.7%)	41 (19.8%)	0.386
Statins	25 (10.5%)	5 (16.7%)	20 (9.7%)	0.243
Metformin	9 (3.8%)	1 (3.3%)	8 (3.9%)	0.887
β‐blocker	42 (17.7%)	6 (20.0%)	36 (17.4%)	0.727

Abbreviations: CUP, cancer of unknown primary; HA(−), no hospital admission registered; HA(+), hospital admission registered; HCC, hepatocellular carcinoma; HNSCC, head and neck squamous cell carcinoma; ICI, immune checkpoint inhibitor; NSCLC, non‐small‐cell lung cancer; RCC, renal cell carcinoma; SCC, squamous cell carcinoma; SCLC, small‐cell lung cancer; UC, urothelial carcinoma; wks, weeks.

^a^ (incl. SCLC, Cutane SCC, HCC, UC, Mesothelioma, CUP, and pleomorphic carcinoma). Bold values as marker for statistical significance (*p* < 0.05).

Treatment in an adjuvant setting showed no imbalance among the subgroups. Nevertheless, the number of prior therapies was increased in the HA(+) patients, while chemotherapy prior to or in addition to ICI was also balanced among the subgroups. Both the type of immune checkpoint inhibitor and the median time until discontinuation of ICI therapy showed statistical differences between the subgroups. The risk of hospitalization was strongly increased in patients with nivolumab/ipilimumab combination therapy, while the risk was similar (avelumab) or lower for all other ICIs. The subgroups had no significant differences in the best objective response (BOR), although the HA(+) cohort showed no complete remissions, fewer partial remissions, and increased primary PD rates. Hepatic, gastrointestinal, endocrine, and renal AEs were significantly more frequent in the HA(+) cohort. However, discontinuation of ICI treatment due to AEs was not increased. The data showed no statistical difference in the use of common comedication during ICI therapy, apart from steroids (Table 2).

**TABLE 2:** Characteristics of immune checkpoint inhibitor therapy of the total ICOG cohort and the subgroups with and without hospital admission (HA).

Parameter	ICOG cohort (*n* = 237)	HA (+) (*n* = 30)	HA (−) (*n* = 207)	*p*
Initial diagnosis to ICI start, median (range), mo	7 (0–561)	17 (0–200)	7 (0–561)	0.776
Treating department, *n* (%)				
Dermatology	142 (59.9%)	23 (76.7%)	119 (57.5%)	0.226
Oncology	57 (24.1%)	5 (16.7%)	52 (25.1%)	
Pulmology	35 (14.8%)	2 (6.7%)	33 (15.9%)	
Gastroenterology	3 (1.3%)	0	3 (1.4%)	
Adjuvant, *n* (%)	79 (33.3%)	8 (26.7%)	71 (34.3%)	0.388
Number of prior therapies, *n* (%)				
0	180 (75.9%)	16 (53.3%)	164 (79.2%)	**0.002**
1	33 (13.9%)	8 (26.7%)	25 (12.1%)	
2	14 (5.9%)	4 (13.3%)	10 (4.8%)	
≥ 3	10 (4.1%)	2 (6.7%)	8 (3.9%)	
CTX prior to ICI, *n* (%)	21 (8.9%)	2 (6.7%)	19 (9.2%)	0.646
CTX in addition to ICI, *n* (%)	36 (15.2%)	2 (6.7%)	34 (16.4%)	0.161
ICI administered *n* (%)				
Nivolumab	109 (46.0%)	9 (30.0%)	100 (48.3%)	**0.001**
Pembrolizumab	57 (24.1%)	4 (13.3%)	53 (25.6%)	
Nivolumab/Ipilimumab	47 (19.8%)	16 (53.3%)	31 (15.0%)	
Atezolizumab	11 (4.6%)	0 (0%)	11 (5.3%)	
Avelumab	7 (3.0%)	1 (3.3%)	6 (2.9%)	
Cemiplimab	3 (1.3%)	0 (0%)	3 (1.4%)	
Durvalumab	2 (0.8%)	0 (0%)	2 (1.0%)	
Ipilimumab	1 (0.4%)	0 (0%)	1 (0.5%)	
Duration of ICI treatment, median (range), d	186.5 (0–966)	69 (0–520)	226 (0–966)	**< 0.001**
Best objective response of ICI, *n* (%)				
CR	7 (3%)	0 (0%)	7 (3.4%)	0.384
PR	43 (18.1%)	4 (13.3%)	39 (18.8%)	
SD	37 (15.6%)	7 (23.3%)	30 (14.5%)	
PD	39 (16.5%)	8 (26.7%)	31 (15%)	
MR	7 (3%)	0 (0%)	7 (3.4%)	
Non‐PD^a^	67 (28.3%)	7 (23.3%)	60 (29%)	
NA	37 (15.6%)	4 (13.3%)	33 (15.9%)	
Adverse events, *n* (%)				
Skin	64 (27%)	12 (40%)	52 (25.1%)	0.086
Neurological	49 (20.7%)	7 (23.3%)	42 (20.3%)	0.700
Gastrointestinal	48 (20.3%)	17 (56.7%)	31 (15%)	**< 0.001**
Endocrine	30 (12.7%)	8 (26.7%)	22 (10.6%)	**0.014**
Muscle	28 (11.8%)	2 (6.7%)	26 (12.6%)	0.350
Joints	20 (8.4%)	4 (13.3%)	16 (7.7%)	0.302
Liver	14 (5.9%)	6 (20%)	8 (3.9%)	**< 0.001**
Respiratory tract	9 (3.8%)	1 (3.3%)	8 (3.9%)	0.887
Cardiovascular	7 (3%)	2 (6.7%)	5 (2.4%)	0.199
Renal	7 (3%)	3 (10%)	4 (1.9%)	**0.015**
Others	27 (11.4%)	2 (6.7%)	25 (12.1%)	0.383
Discontinuation due to AE, *n* (%)	70 (29.5%)	7 (23.3%)	63 (30.4%)	0.426
Comedication during ICI, *n* (%)				
Steroids	70 (29.5%)	26 (86.7%)	44 (21.3%)	**< 0.001**
Antibiotics	6 (2.5%)	1 (3.3%)	5 (2.4%)	0.765
PPI	58 (24.5%)	9 (30.0%)	49 (23.7%)	0.451
Statins	23 (9.7%)	5 (16.7%)	18 (8.7%)	0.168
Metformin	10 (4.2%)	1 (3.3%)	9 (4.3%)	0.796
Beta‐blocker	48 (20.3%)	7 (23.3%)	41 (19.8%)	0.653

Abbreviations: CR, complete remission; CTX, chemotherapy; d, days; HA(−), no hospital admission registered; HA(+), hospital admission registered; ICI, immune checkpoint inhibitor; mo, months; MR, mixed response; NA, not applicable; PD, progressive disease; PPI, proton pump inhibitor; PR, partial remission; SD, stable disease.

^a^ Patients in an adjuvant therapy setting. Bold values as marker for statistical significance (*p* < 0.05).

### 3.2 Characteristics of the Inpatient Stay

Thirty of the 237 patients (12.6%) were hospitalized. The median time in therapy up to hospital admission was 66.5 (3–576) days. Dual PD‐1/CTLA‐4 inhibition with Nivolumab/Ipilimumab were 68 (15–257) days and ICI monotherapy with Nivolumab, Pembrolizumab, or Avelumab were 74 (3–576) days in therapy until hospitalization occurred (Figure S3). These results did not differ significantly between these groups (*p* = 0.371). The disease distribution in HA(+) and HA(−) did not differ significantly (*p* = 0.202), however, malignant melanomas were slightly more predominant among HA(+) patients, whereas NSCLC and HNSCC tended to be underrepresented in the HA(+) cohort. A high percentage of patients were already metastatic at initial diagnosis (*n* = 22, 73.3%). At the onset of ICI‐therapy, 24 patients had a metastasized disease (*n* = 24, 80%). Half of the HA(+) patients had prior cardiovascular disease. The largest proportion received dual checkpoint blockade with nivolumab and ipilimumab. The median duration of the inpatient stay was 7 (1–34) days. The most common reason for hospital admission was colitis (*n* = 8, 26.7%), followed by hypophysitis (*n* = 4, 13.3%), pancreatitis‐ and immune‐mediated polyneuropathy (*n* = 2, 6.7%). Hepatitis, gastritis, encephalitis, nephritis, and pneumonitis occurred in one patient each (*n* = 1, 3.3%), and other irAEs were observed in 13.3% of cases (*n* = 4, 13.3%). Other tumor‐associated complications, including heart failure, tumor progression, dyspnea, epileptic seizure, and a femoral shaft fracture, accounted for the remaining 16.7% (*n* = 5). Two patients with pancreatitis and one with hypophysitis were admitted with additional colitis, which was not the primary cause of admission. The majority of AEs were CTCAE grade 3 (*n* = 15, 50%), ahead of grade 2 (*n* = 6, 20%). Grade 1 (*n* = 1, 3.3%) and grade 4 AEs (*n* = 2, 6.7%) were rarer. Most of the admissions were treated without intensive care unit (ICU) support (*n* = 23, 76.7%). Three patients were admitted to intermediate care unit (IMC, 10%) and 2 to ICU (6.6%). Thirteen of the 30 hospitalized patients (43.3%) received invasive diagnostics. Colonoscopy was most common (*n* = 9, 30%), followed by spinal tap (*n* = 4, 13.3%) and gastroscopy (*n* = 1, 3.3%). One patient with hypophysitis underwent colonoscopy for a colitis that did not require hospitalization. A variety of consultations were conducted during the inpatient stays: gastroenterology (*n* = 14, 46.7%). Neurology (*n* = 8, 26.7%), psychosomatic (*n* = 7, 23.3%), ophthalmology, nephrology, and radiotherapy (each *n* = 5, 16.7%). The inpatient stay resulted in an improved condition for most patients (*n* = 23, 76.7%), one patient's adverse event resolved completely (3.3%), three did not improve (10%) and one patient died (due to progression and multi‐organ failure; 3.3%) (Table 3).

**TABLE 3:** Characteristics of hospital admission (HA).

Parameter	HA (+)
Hospitalization under ICI, *n* (% of total cohort)	30 (12.6%)
Time in therapy up to HA (+), median (range), d	66.5 (0–520)
Duration of inpatient stay, median (range), d	7 (1–34)
Reason for hospital admission, *n* (% of HA(+) cohort)	
Colitis	8 (26.7%)
Hypophysitis	4 (13.3%)
Pancreatitis	2 (6.7%)
PNP	2 (6.7%)
Encephalitis	1 (3.3%)
Pneumonitis	1 (3.3%)
Hepatitis	1 (3.3%)
Gastritis	1 (3.3%)
Nephritis	1 (3.3%)
Other irAE^a^	4 (13.3%)
Tumor‐associated complications^b^	5 (16.7%)
CTCAE, *n* (% of HA(+) cohort)	
1	1 (3.3%)
2	6 (20%)
3	15 (50%)
4	2 (6.7%)
NA^c^	6 (20%)
Type of inclinic support, *n* (% of HA(+) cohort)	
General	23 (76.7%)
IMC	3 (10%)
ICU	2 (6.7%)
Multi‐organ failure, *n* (% of HA(+) cohort)	1 (3.3%)
Invasive diagnostics, *n* (% of HA(+) cohort)	
Colonoscopy^d^	9 (30%)
Lumbar puncture	4 (13.3%)
Gastroscopy	1 (3.3%)
Outcome, *n* (% of HA(+) cohort)	
Resolved	1 (3.3%)
Improved	23 (76.7%)
Not improved	3 (10%)
Death	1 (3.3%)
Consults, *n* (% of HA(+) cohort)	
Gastroenterology	14 (46.7%)
Neurology	8 (26.7%)
Psychosomatics	7 (23.3%)
Nephrology	5 (16.7%)
Ophthalmology	5 (16.7%)
Radiotherapy	5 (16.7%)
Cardiology	4 (13.3%)
Dermatology	3 (10%)
Oncological nursing	2 (6.7%)
Pulmonology	2 (6.7%)
Surgery	2 (6.7%)
Urology	2 (6.7%)
Otorhinolaryngology	1 (3.3%)

Abbreviations: d, days; HA(+), hospital admission registered; ICI, immune checkpoint inhibitor; ICU, intensive care unit; IMC, intermediate care unit; irAE, immune‐related adverse event; PNP, polyneuropathy.

^**a**^ Incl. myositis‐myasthenia overlap syndrome, autoimmune thyreopathy, dopple vision, cytokine‐release syndrome.

^**b**^ Incl. heart insufficiency, tumor progression, dyspnea, epileptic seizure, and femoral shaft fracture.

^c^ Grading was not determined during hospitalization.

^d^ Four had endoscopic biopsies.

### 3.3 Efficacy and Outcome of ICI‐Treated Patients in Dependence of Hospitalization

There were no significant differences in overall response rates, although tendencies that hospitalized patients were associated with shortened PFS and prolonged OS were observed.

Regarding the analysis of PFS, median time was 14.5 months (95% CI: 3.6–25.5 months) for the HA(−) group and 5.8 months (95% CI: 4.4–7.3 months) for the HA(+) group (HR: 1.402 (95% CI: 0.823–2.388), log‐rank *p* = 0.211). Median OS in both groups was not reached (HR 0.432 (95% CI: 0.103–1.808), log‐rank *p* = 0.237).

**FIGURE 1:**
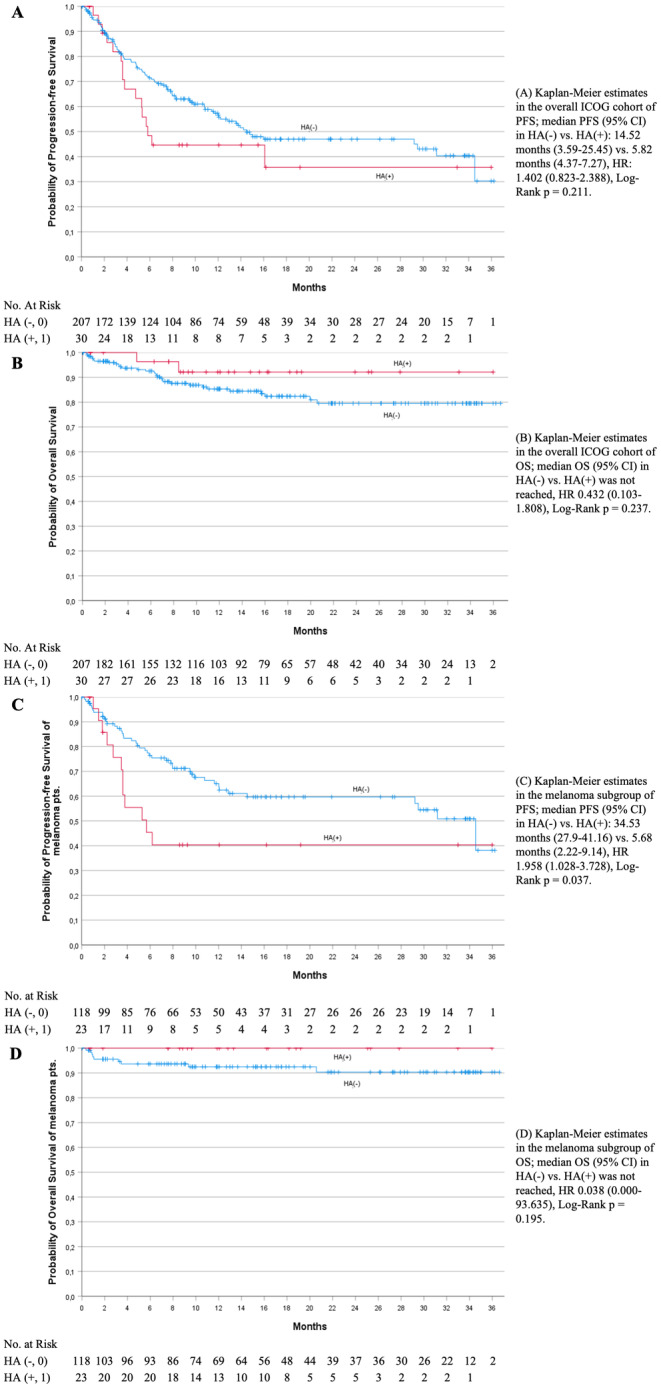
Progression‐free survival (PFS) and overall survival (OS) in dependence of hospitalization within the total ICOG cohort and the melanoma subgroup. (A) Kaplan–Meier estimates in the overall ICOG cohort of PFS; median PFS (95% CI) in HA(−) vs. HA(+): 14.52 months (3.59–25.45) vs. 5.82 months (4.37–7.27), HR: 1.402 (0.823–2.388), log‐rank *p* = 0.211. (B) Kaplan–Meier estimates in the overall ICOG cohort of OS; median OS (95% CI) in HA(−) vs. HA(+) was not reached, HR 0.432 (0.103–1.808), log‐rank *p* = 0.237. (C) Kaplan–Meier estimates in the melanoma subgroup of PFS; median PFS (95% CI) in HA(−) vs. HA(+): 34.53 months (27.9–41.16) vs. 5.68 months (2.22–9.14), HR 1.958 (1.028–3.728), log‐rank *p* = 0.037. (D) Kaplan–Meier estimates in the melanoma subgroup of OS; median OS (95% CI) in HA(−) vs. HA(+) was not reached, HR 0.038 (0.000–93.635), log‐rank *p* = 0.195. HA(−), no hospital admission registered; HA(+), hospital admission registered; HR, hazard ratio; mo, months; OS, overall survival; PFS, progression‐free survival.

A subgroup analysis of malignant melanoma patients supported the observation of a worsened PFS trend in the HA(+) group, with significant findings indicating a median PFS of 5.7 months (95% CI: 2.2–9.1) compared to 34.5 months (95% CI: 27.9–41.2) in the HA(−) group [HR 1.958 (95% CI: 1.028–3.728); log‐rank *p* = 0.037]. The median OS for malignant melanoma patients was also not reached [HR 0.038 (95% CI: 0–93.635); log‐rank *p* = 0.195] (Figure 1).

### 3.4 Patient, Tumor, and Treatment Characteristics as Risk Predictors for Hospitalization

The univariate analysis showed significance for the following parameters: pulmonary metastasis vs. none, cerebral metastasis vs. none, Nivolumab/Ipilimumab combination therapy vs. other therapies, and number of therapies before ICI treatment.

**FIGURE 2:**
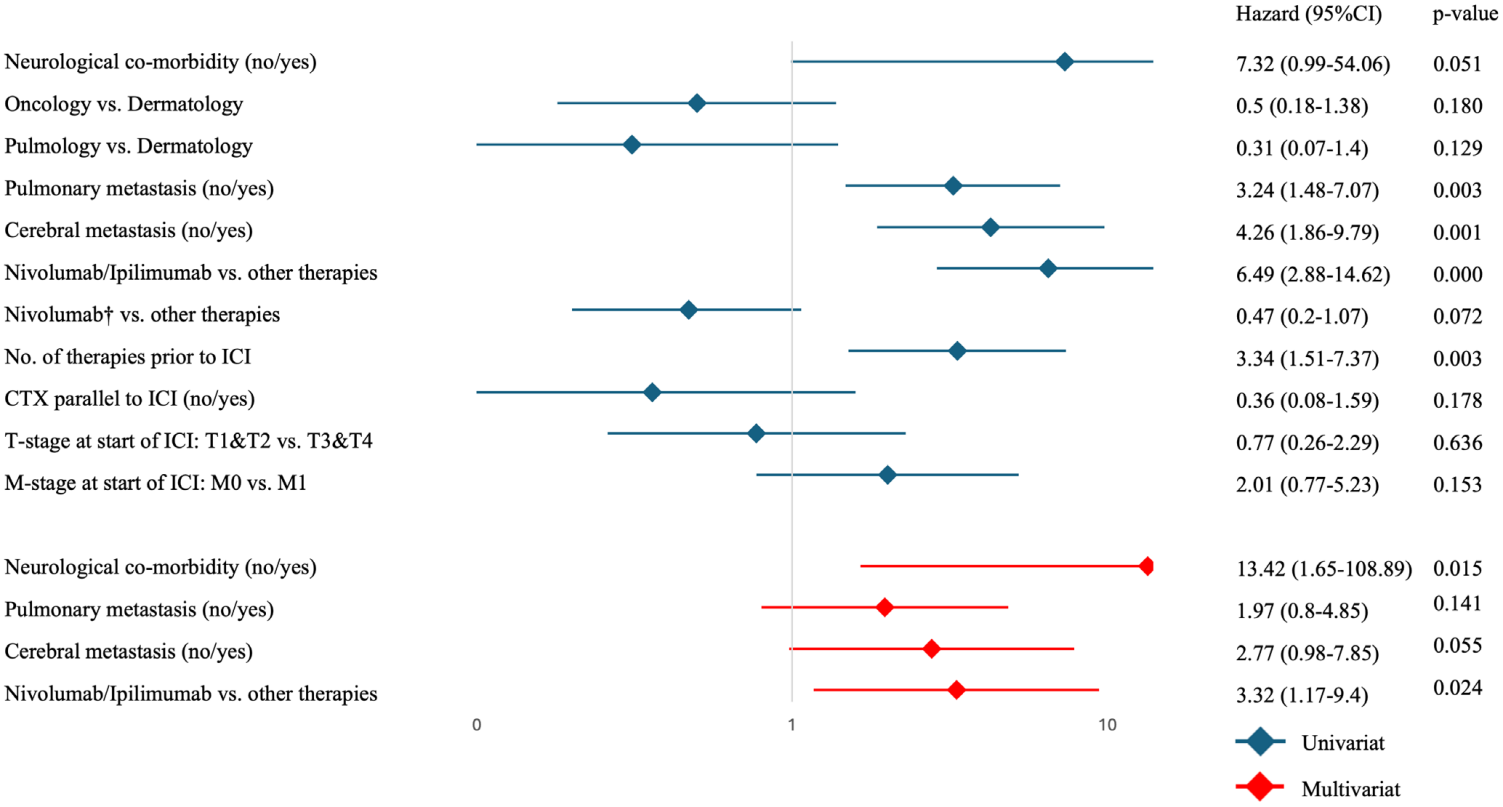
Risk factor analysis for hospitalization by univariate and multivariate analyses (UVA and MVA). CTX, chemotherapy; HA(+), hospital admission registered; ICI, immune checkpoint inhibitor; *only as the single ICI in a regimen.

In subsequent multivariate analysis, including neurological comorbidity, pulmonary metastasis, cerebral metastasis, and Nivolumab/Ipilimumab combination therapy vs. other therapies both, neurological comorbidity and Nivolumab/Ipilimumab combination therapy as independent risk factors for hospital admission could be confirmed (Figure 2).

### 3.5 Laboratory Findings in Dependence of Hospitalization

Regarding correlative analyzes on laboratory tests, significant differences in c‐reactive protein (CRP) at baseline with lower values in HA(+) patients could be detected, although CRP was significantly higher in the HA(+) group at the first visit. Creatine kinase (CK) at V1‐2 and at EOS was found to be significantly lower in the HA(+) patients. Furthermore, albumin levels at baseline visit were significantly higher in the HA(+) group. The remaining parameters were not of special interest (Table S2).

Univariate analysis testing laboratory parameters as potential risk factors for hospitalization showed significance for the following parameters (Table S3):

Higher absolute Troponin T (TnT) at V3 as well as at V4, higher absolute albumin at baseline, and higher absolute ferritin at V4 resulted in a higher risk for hospitalization.

Higher absolute CK at V3 as well as at EOS, higher absolute hemoglobin at EOS, lower absolute neutrophils at V3, lower absolute neutrophils at V4, and lower absolute neutrophils at EOS resulted in a lower risk of hospitalization.

However, multivariate analysis could not confirm any independent risk factors regarding laboratory parameters.

## 4 Discussion

Our study provides first insights into the hospital admission patterns and outcomes of patients undergoing ICI therapy for various solid malignancies. The findings shed light on the clinical characteristics, management strategies, and outcomes of irAEs necessitating inpatient diagnostics and treatment in this patient population.

To date, only a few publications have compiled the frequency and details of hospital admissions in the context of ICI therapy, in particular due to irAEs [20, 21, 22, 23]. To the best of our knowledge, no prospective observational study cohort has yet been analyzed regarding hospitalization under ICI therapy.

Overall, our cohort shows comparable results with previous reports in terms of age and gender as well as the distribution of ICI therapies [20, 21, 22, 23]. Our total cohort includes a higher proportion of 59.5% melanoma patients, which is most likely due to a high recruitment rate in the Department of Dermatology. In addition, malignant melanoma was the most common entity for ICI as standard of care during the recruitment period [24].

Compared to previous publications, our data report a cohort of 237 patients with annotated information regarding clinical parameters, specifically neurological side effects, and a structured collection of accompanying laboratory parameters [17, 25].

The hospital admission rate of 12.7% is in line with the literature. The majority of hospitalizations were due to irAEs (25/30 cases, 83.3%), whereas other publications have described significantly higher hospitalization rates with comparable incidences of irAEs [21, 22]. Comparable to the findings of Balaji et al. and Ahern et al., the majority of HA(+) patients showed high grade (≥ grade 3) irAEs. The median duration of hospitalization of 7 days is comparable to previously described cohorts [20, 23].

The data set presented here provides evidence for the presence of pulmonary and cerebral metastases as potential independent risk factors for hospital admission. The strongest risk factor for the occurrence of irAE was the implementation of a dual checkpoint blockade, in line with existing evidence in the literature [16, 20, 26].

Compared to the literature, our cohort exhibited a higher proportion of colitis (26.7%) and hypophysitis (13.3%). This may indicate that detailed knowledge of rare ICI side effects is helpful for timely action. Moreover, regular use of corticosteroids was described, whereas no other immunosuppressants were given in the treatment of the observed irAEs.

Laboratory analysis regarding inflammatory parameters and cardiological parameters were investigated. Even though the comparison of the inflammation parameters between the HA(+) group and the HA(−) group did not show any strong differences, there was a trend toward increasing inflammation parameters (CRP, leukocytes, absolute neutrophil count) in the first months of treatment up to the V4 visit (Table S2). This trend was not observed in the HA(−) group. These results are consistent with the median time of 66.5 days in therapy to hospitalization due to irAEs (time point). At the time of EOS, the laboratory parameters returned to normal, indicating that the immune‐mediated side effects had resolved.

The results of the time to hospitalization depending on the ICI regimen used can only be evaluated to a limited extent due to the small subgroup numbers (Figure S3). The median time of admission for patients treated with dual ICI (median time of admission 68 (15–257) days) tends to be earlier than the median time of admission for PD‐1‐ and PD‐L1‐directed monotherapy, which is in line with current literature [27].

Regarding the clinical outcome of the tumor disease, no significant association on PFS or OS in the hospitalized cohort could be observed. Within the literature, a better clinical outcome has been described in the presence of manageable irAEs [28, 29, 30, 31, 32, 33]. Overall, our data suggest that even irAEs leading to hospital admission can be well managed in most cases due to timely diagnosis and treatment and do not lead to a worse clinical outcome. The data show a trend toward an improved OS in the HA(+). The only death in the present cohort was not due to the consequences of irAEs, but to the progression of the underlying disease. These results will be updated when the median OS in the cohort is reached and the data are more mature.

This data particularly emphasizes the need for interdisciplinary care. In 14 cases, invasive examinations were required, consistent with prior reports [20].

Due to the risk profile of ICI, the possibility of collaboration with a maximum care center is useful, although, in principle, ICI can be performed at institutions of primary and secondary care (e.g., office‐based oncologists). This report confirms the advantages of interdisciplinary treatment and collaboration between referring physicians and maximum care providers (e.g., academic centers).

Limitations of our study include the single‐center setting, the limited number of cases, and the short follow‐up. Due to the high number of newly approved drugs, especially novel combination therapies (e.g., dual checkpoint blockade, ICI/TKI combinations, or combinations of ICI and chemotherapy), the reported cohort does not completely reflect the current landscape of immuno‐oncology.

Overall, no hospital readmissions due to irAEs were reported in our cohort during the observation period. An updated follow‐up will provide insights into the long‐term tolerability of ICI following the occurrence of higher‐grade irAEs. Due to the ongoing collection of data, a reliable comparison between irAEs and non‐irAEs could be possible in future analyses, including the identification of predisposing factors and biomarkers for the occurrence of irAEs.

## 5 Conclusion

In conclusion, our study provides first insights into the hospitalization patterns, management strategies, and outcomes of irAEs in ICI therapy. The hospitalization rate underscores the need for vigilant monitoring, especially in gastrointestinal and endocrine toxicities. Our findings highlight the need for interdisciplinary collaboration in the management of immune‐related toxicities and emphasize the importance of timely recognition. Specifically, patients with dual ICI, cerebral or pulmonary metastasis, and neurological comorbidities may benefit from appropriate management to achieve optimized treatment outcomes. With a low hospitalization rate, short stays, and improved outcomes, our data support the outpatient administration of ICI. Further research is needed to understand irAEs mechanisms and develop mitigation strategies.

## Author Contributions

**Jonas Paul Wiegmann:** writing – original draft; conceptualization; investigation; writing – review and editing; visualization; validation; methodology; formal analysis; data curation. **Tabea Fröhlich:** writing – original draft; conceptualization; investigation; methodology; validation; visualization; writing – review and editing; formal analysis; data curation; project administration. **Nora Möhn:** funding acquisition; writing – review and editing. **Laura Duzzi:** investigation; writing – review and editing. **Emily Narten:** investigation; writing – review and editing. **Johanna Aurich:** investigation; writing – review and editing. **Janin Thomas:** investigation; writing – review and editing. **Lea Grote‐Levi:** investigation; writing – review and editing. **Susann Mahjoub:** investigation; writing – review and editing. **Dominik Berliner:** writing – review and editing; resources. **Thomas Wirth:** writing – review and editing; Resources. **Heiko Golpon:** resources; writing – review and editing. **Benjamin‐Alexander Bollmann:** writing – review and editing; resources. **Imke Von Wasilewski:** funding acquisition; Writing – review and editing; Resources. **Ralf Gutzmer:** funding acquisition; Writing – review and editing; Resources. **Florian H. Heidel:** writing – review and editing; resources. **Thomas Skripuletz:** funding acquisition; writing – review and editing; resources. **Gernot Beutel:** funding acquisition; writing – review and editing; resources; supervision. **Philipp Ivanyi:** funding acquisition; writing – review and editing; resources; supervision.

## Ethics Statement

The study was approved by the ethics committee of Hannover Medical School, vote number 2413–2014. Analyses were done in concordance with local ethic committee recommendations respecting the Declaration of Helsinki in its latest version.

## Conflicts of Interest

J.P.W., T.F., N.M., L.D., E.N., J.A., L.G.L., S.M., J.T., B.‐A.B., D.B., T.W., H.G., F.H.H., and T.S.: none. I.V.W.: Honoraria–BMS Brazil; Kyowa Kirin; MSD; Novartis; Sanofi; Stemline Therapeutics. Consulting or Advisory Role–Bristol‐Myers Squibb; MSD; Sanofi. Travel, Accommodations, Expenses–Bristol‐Myers Squibb; Kyowa Kirin; MSD; Novartis; Sanofi; Stemline Therapeutics. R.G.: Honoraria–BMS, MSD, Novartis, Amgen, Merck Serono, Almirall Hermal, SUN, Sanofi/Regeneron, Pierre‐Fabre. Consulting or Advisory Role–BMS, Novartis, Almirall Hermal, MSD, Amgen, SUN, Sanofi/Regeneron, Pierre‐Fabre, 4SC, MerckSerono, Pfizer, Immunocore, Delcath. Travel, Accommodations, Expenses–SUN, Pierre‐Fabre, Boehringer Ingelheim. Research Grant (to institution)–Amgen, Merck‐Serono, SUN Pharma, Sanofi/Regeneron, Kyowa‐Kirin, Almirall‐Hermal. G.B.: Consulting or Advisory Role–Shionogi. Speakers' Bureau–Jazz Pharmazeuticals; MSD. P.I.: Stock and Other Ownership Interests–BB Biotech Ventures. Honoraria–AIMM Therapeutics; AstraZeneca; Bayer/Vital; Bristol‐Myers Squibb; Eisai; EUSA Pharma; Ipsen; Merck Serono; MSD Oncology; Roche Pharma AG. Consulting or Advisory Role–Bayer/Vital; Bristol‐Myers Squibb; ClinSol; Deciphera; Eisai; EUSA Pharma; Ipsen; Merck Serono; MSD; Pfizer. Travel, Accommodations, Expenses–Bristol‐Myers Squibb; Ipsen; Merck Serono. Other Relationship–Merck Serono; Pfizer.

## Supporting information


Data S1.

## Data Availability

The data sets used and/or analyzed during the current study are available from the corresponding author upon reasonable request.
