# Supplementary material for: Characterization of Hospital Admissions During Immune Checkpoint Inhibitor Therapy: Insights From the ICOG Study
Source: Cancer Med. 2025 Jan 25;14(3):e70582. doi: 10.1002/cam4.70582 (PMC11761430; doi:10.1002/cam4.70582)
Supplement: Supplementary file 1 — Data S1. [file CAM4-14-e70582-s001.docx]

**Title**

# Characterization of Hospital Admissions During Immune Checkpoint Inhibitor Therapy: Insights from the ICOG Study

**Authors**

Wiegmann JP^1,2^**^†^**, Fröhlich TC^1,2^**^†^**, Möhn N^2,4^, Duzzi L^4^, Narten E^4^, Aurich J^4^, Grote-Levi L^2,4^, Mahjoub S^4^, Berliner D^2,5^, Wirth T^2,6^, Golpon H^2,7^, Bollmann BA^2,7^, Von Wasilewski I^2,8^, Gutzmer R^9^, Heidel FH^1,2^, Skripuletz T^2,4^, Beutel G^1,2^**^†^** and Ivanyi P^1,2,3^**^†^**; on behalf of ICOG-CCCH (Immune Cooperative Oncology Group; Comprehensive Cancer Center Hannover)

**Supplemental Material**

**Supplemental Figure 1:** Study design.

**Supplemental Figure 2:** Consort diagram. Displayed is the inclusion process of hospitalized (HA(+)) and immune checkpoint inhibitor-treated patients under investigation during the observation period 11/2019 - 12/2022 within the ICOG-Study.

**Supplemental Table 1:** Patient characteristics at initial diagnosis of the overall ICOG cohort and the subgroups with and without hospital admission (HA).

**Supplemental Table 2:** Laboratory parameters of the overall ICOG cohort and the subgroups with and without hospital admission (HA).

**Supplemental Table 3:** Univariate and multivariate analysis of laboratory parameters of the overall ICOG cohort.

Supplemental Material

**Supplemental Figure 1:** Study design


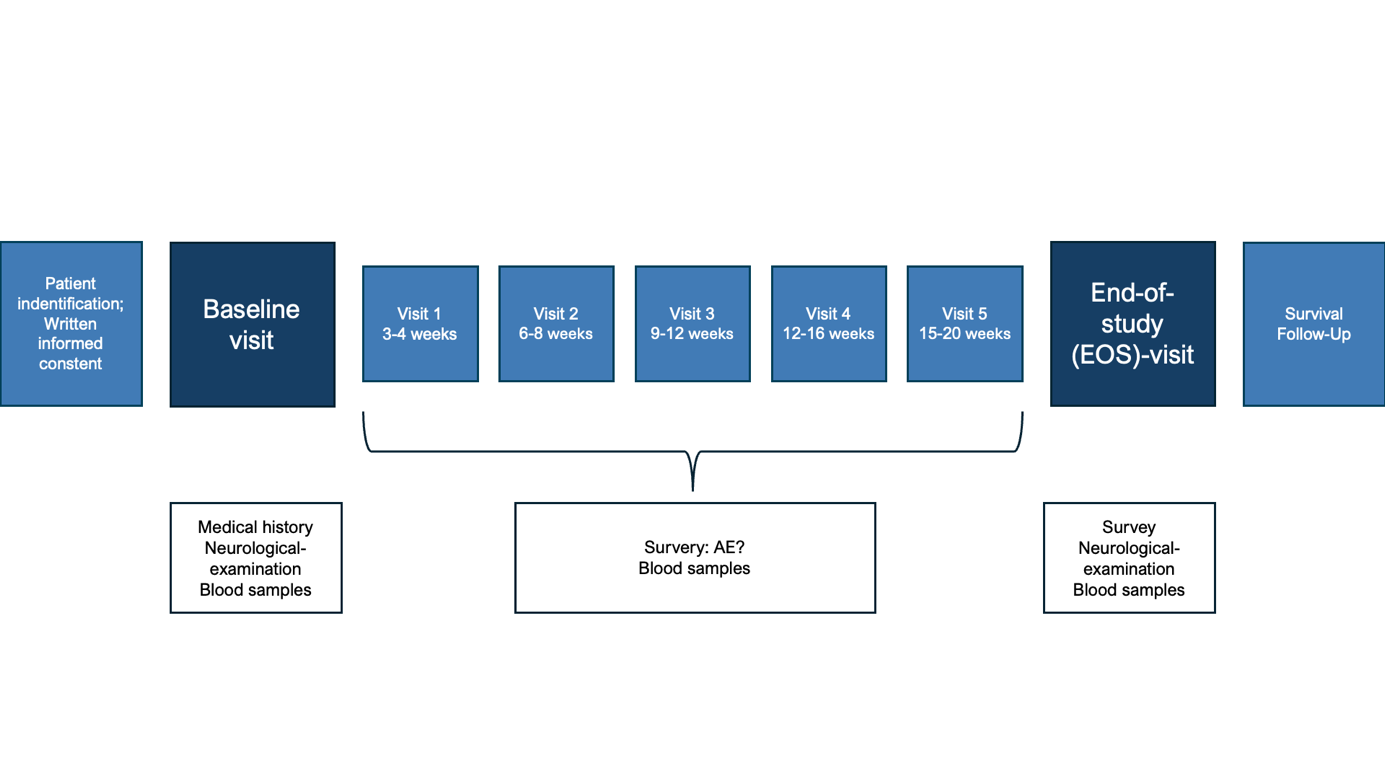


## **Supplemental Figure 2:** Consort diagram. Displayed is the inclusion process of hospitalized (HA(+)) and immune checkpoint inhibitor-treated patients under investigation during the observation period 11/2019 - 12/2022 within the ICOG-Study.


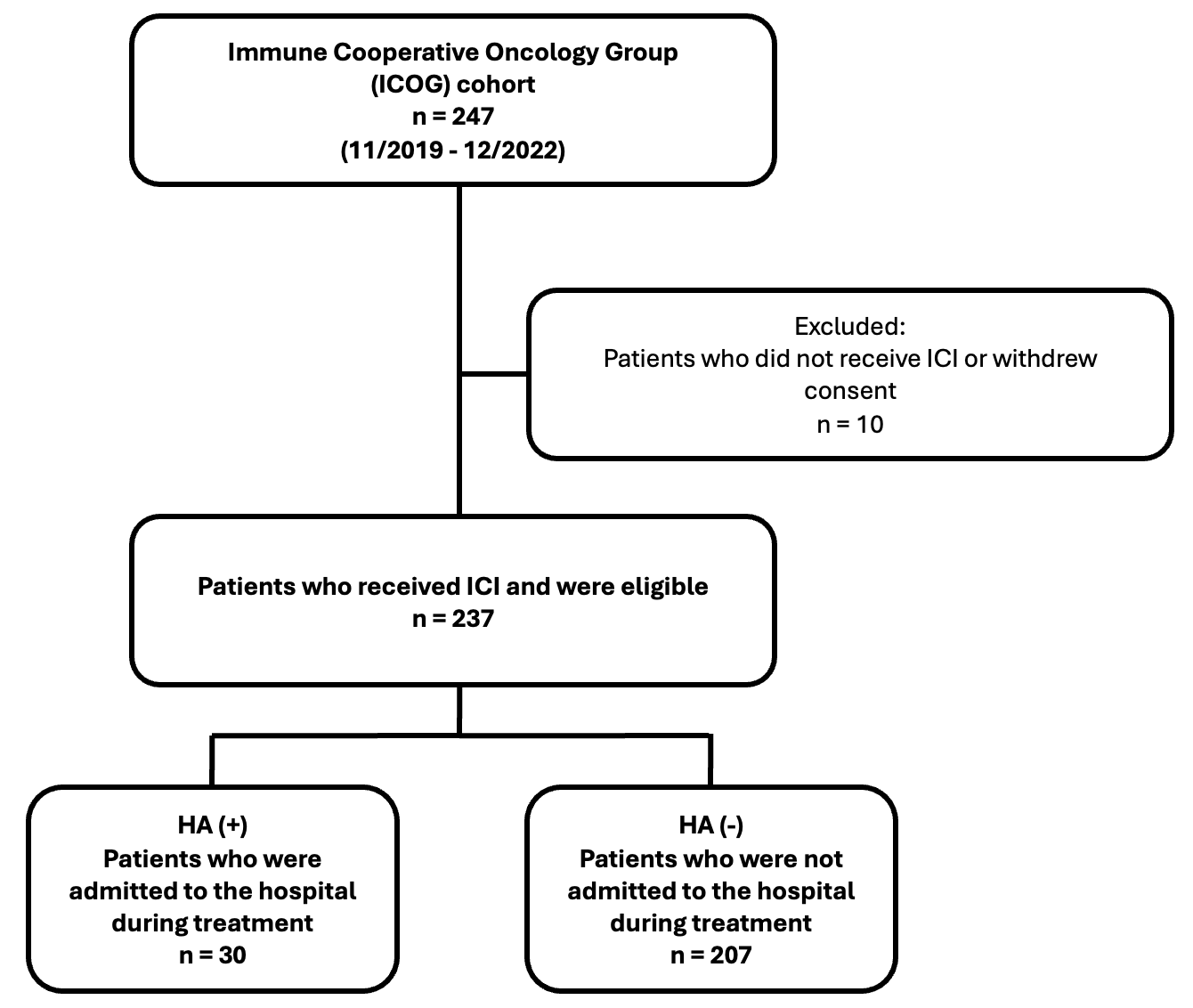


## Abbreviations: HA(-), no hospital admission registered; HA(+), hospital admission registered; ICOG, Immune Cooperative Oncology Group; ICI, immune checkpoint inhibitor.

**Supplemental Figure 3:** Time from start of therapy to hospital admission depending on the therapy regimen immune checkpoint inhibitor mono- or dual therapy. Median, mean and range are shown.


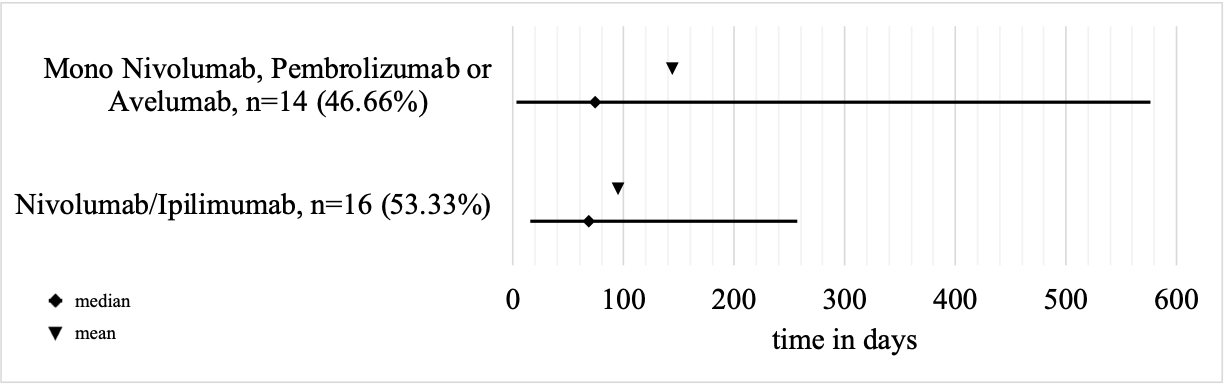


**Supplemental** **Table 1:** Patient characteristics at initial diagnosis of the overall ICOG cohort and the subgroups with and without hospital admission (HA).

| Parameter | **ICOG Cohort**  **(n = 237)** | **HA (+)**  **(n = 30)** | **HA (-)**  **(n = 207)** | **p-value** |
| --- | --- | --- | --- | --- |
| Age at primary diagnosis, Median (Range), years | 62 (20-85) | 59.5 (47-85) | 62 (20-85) | 0.537 |
| Sex, n (%) |  |  |  | 0.571 |
| Male | 155 (65.4%) | 21 (70%) | 134 (64.7%) |  |
| Female | 82 (34.6%) | 9 (30%) | 73 (35.3%) |  |
| Solid neoplasia, n (%) |  |  |  | 0.202 |
| Malignant Melanoma | 141 (59.5%) | 23 (76.7%) | 118 (57.0%) |  |
| RCC | 31 (13.1%) | 4 (13.3%) | 27 (13.0%) |  |
| NSCLC | 30 (12.7%) | 2 (6.7%) | 28 (13.5%) |  |
| HNSCC | 18 (7.6%) | 1 (3.3%) | 17 (8.2%) |  |
| Other^†^ | 17 (7.2%) | 0 | 17 (8.2%) |  |
| T-stage, n (%) |  |  |  | 0.945 |
| T1-T2 | 47 (19.8%) | 6 (20.0%) | 41 (19.8%) |  |
| T3-T4 | 91 (38.4%) | 12 (40.0%) | 79 (38.2%) |  |
| Tx | 99 (41.8%) | 12 (40.0%) | 87 (42.0%) |  |
| N-stage, n (%) |  |  |  | 0.357 |
| N0 | 61 (25.7%) | 9 (30.0%) | 52 (25.1%) |  |
| N+ | 119 (50.2%) | 12 (40.0%) | 107 (51.7%) |  |
| Nx | 57 (24.1%) | 9 (30.0%) | 48 (23.2%) |  |
| M-stage, n (%) |  |  |  | 0.327 |
| M0 | 122 (51.5%) | 13 (43.3%) | 109 (52.7%) |  |
| M1 | 71 (30.0%) | 11 (36.7%) | 60 (29.0%) |  |
| Mx | 44 (18.6%) | 6 (20.0%) | 38 (18.4%) |  |
| Nicotine abuse, n (%) |  |  |  | 0.852 |
| no | 54 (22.4%) | 8 (26.7%) | 45 (21.7%) |  |
| yes | 26 (11%) | 3 (10%) | 23 (11.1%) |  |
| history of | 34 (14.3%) | 3 (10%) | 31 (15%) |  |
| N/A | 124 (52.3%) | 16 (53.3%) | 108 (52.2%) |  |
| Alcohol abuse, n (%) |  |  |  | 0.548 |
| no | 83 (35%) | 11 (36.7%) | 72 (34.8%) |  |
| yes | 8 (3.4%) | 0 | 8 (3.9%) |  |
| history of | 8 (3.4%) | 1 (3.3%) | 7 (3.4%) |  |
| N/A | 138 (58.2%) | 18 (60%) | 120 (58%) |  |
| PD-L1-status, n (%) |  |  |  | 0.185 |
| Positive | 48 (20.3%) | 3 (10%) | 45 (21.7%) |  |
| Negative | 6 (2.5%) | 0 | 6 (2.9%) |  |
| N/A | 183 (77.2%) | 27 (90%) | 156 (75.4%) |  |

Abbreviations: HA(-), no hospital admission registered; HA(+), hospital admission registered; HCC, hepatocellular carcinoma; HNSCC, head and neck squamous cell carcinoma; ICI, immune checkpoint inhibitor; NSCLC, non-small cell lung cancer; RCC, renal cell carcinoma; SCC, squamous cell carcinoma; SCLC, small cell lung cancer; UC, urothelial carcinoma; CUP, cancer of unknown primary; wks, weeks; **^†^** (incl. CUP and pleomorphic carcinoma)

**Supplemental Table 2:** Laboratory parameters of the overall ICOG cohort and the subgroups with and without hospital admission (HA).

| Parameter | **ICOG cohort**  **(n = 237)** | **HA (+)**  **(n = 30)** | **HA (-) (n = 207)** | **p-value** |
| --- | --- | --- | --- | --- |
| Median CRP (range) in mg/l | | |  |  |
| Baseline | 4.9 (0.5-225) | 4 (0.6-77) | 5.4 (0.5-225) | **0.019** |
| V1 | 5.9 (0.22-290.7) | 6.1 (0.6-52.4) | 5.9 (0.22-290.7) | **0.003** |
| V3 | 4.75 (0.6-347.9) | 6.7 (0.6-91.9) | 4.6 (0.6-347.9) | 0.785 |
| V4 | 6.4 (0.5-183.2) | 13.95 (0.6-58.5) | 4.9 (0.5-183.2) | 0.292 |
| EOS | 4 (0.6-215.3) | 8.8 (0.6-129) | 3.65 (0.6-215.3) | 0.319 |
| Median total leukocytes (range) in tsd/µl | | |  |  |
| Baseline | 7.5 (0.3-33.3) | 6.45 (3.5-10.1) | 7.6 (0.3-33.3) | **0.029** |
| V1 | 7.2 (1-20.5) | 6.8 (3.5-20.5) | 7.3 (1-20.3) | 0.315 |
| V3 | 7 (2.6-24.7) | 7.2 (3.4-16.8) | 7 (2.6-24.7) | 0.157 |
| V4 | 6.6 (2.7-18.4) | 9 (4-15.4) | 6.3 (2.7-18.4) | 0.096 |
| EOS | 6.8 (2.4-24.9) | 7.6 (4.1-24.9) | 6.75 (2.4-23) | **0.048** |
| Neutrophils in normal range, n (% total; % applicable) | | |  |  |
| Baseline | 164 (69.2%; 84.1%) | 22 (73.3%; 95.7%) | 142 (68.6%; 82.6%) | 0.107 |
| V1 | 117 (49.4%; 81.3%) | 15 (50%; 83.3%) | 102 (49.3%; 81%) | 0.809 |
| V3 | 110 (46.4%; 85.3%) | 16 (53.3%; 72.7%) | 94 (45.4%; 87.9%) | 0.068 |
| V4 | 45 (19%; 41.3%) | 6 (20%; 40%) | 52 (25.1%; 67.5%) | **0.043** |
| EOS | 114 (48.1%; 86.4%) | 15 (50%; 78.9%) | 99 (47.8%; 87.6%) | 0.309 |
| Median absolute neutrophils (range) in tsd/µl | | |  |  |
| Baseline | 4.92 (0.19-84.5) | 4.3 (2.14-84.5) | 5.09 (0.19-80.1) | 0.664 |
| V1 | 4.73 (0.79-18.67) | 4.66 (2.03-11.27) | 4.76 (0.79-18.67) | 0.775 |
| V3 | 4.66 (1.29-21.11) | 6.005 (3.13-14.41) | 4.625 (1.29-21.11) | **0.041** |
| V4 | 4.07 (0.84-12.7) | 6.77 (2.09-12.7) | 3.885 (0.84-11.65) | **0.024** |
| EOS | 4.635 (1.28-21.23) | 5.72 (2.21-21.23) | 4.45 (1.28-21) | 0.073 |
| Median proportional eosinophils (range) in % | | |  |  |
| Baseline | 1.7 (0-66.9) | 1.45 (0.1-9.9) | 1.8 (0-66.9) | 0.491 |
| V1 | 2.2 (0-23.9) | 1.7 (0.6-23.8) | 2.3 (0-23.9) | 0.456 |
| V3 | 2 (0-27.8) | 1.3 (0-9.1) | 2.1 (0-27.8) | 0.126 |
| V4 | 2.4 (0-14.4) | 1.85 (0.1-8) | 2.4 (0-14.4) | 0.500 |
| EOS | 2.1 (0-20.7) | 0.6 (0-7.3) | 2.4 (0-20.7) | **0.001** |
| Median absolute eosinophils (range) in tsd/µl | | |  |  |
| Baseline | 0.14 (0-4.1) | 0.105 (0.01-0.65) | 0.14 (0-4.1) | 0.377 |
| V1 | 0.14 (0-2.3) | 0.13 (0.03-1.7) | 0.15 (0-2.3) | 0.657 |
| V3 | 0.14 (0-0.98) | 0.09 (0-0.33) | 0.155 (0-0.98) | 0.060 |
| V4 | 0.15 (0-1.08) | 0.14 (0.01-0.42) | 0.16 (0-1.08) | 0.395 |
| EOS | 0.15 (0-1.24) | 0.06 (0-0.4) | 0.17 (0-1.24) | **<0.001** |
| Median absolute lymphocytes (range) in tsd/µl | | |  |  |
| Baseline | 1.25 (0.1-116.3) | 1.13 (0.43-34.6) | 1.295 (0.1-116.3) | 0.788 |
| V1 | 1.215 (0.16-23.6) | 1.22 (0.6-2.65) | 1.21 (0.16-23.6) | 0.669 |
| V3 | 1.215 (0.09-35.6) | 1.07 (0.26-35.6) | 1.29 (0.09-30.1) | 0.447 |
| V4 | 1.205 (0.18-14.6) | 1.285 (0.49-14.6) | 1.195 (0.18-5.7) | 0.452 |
| EOS | 1.235 (0.2-23.4) | 1.18 (0.3-11.3) | 1.25 (0.2-23.4) | 0.572 |
| Median creatine kinase (range) in U/l | |  |  |  |
| Baseline | 75 (15-649) | 72 (23-617) | 76 (15-649) | 0.673 |
| V1 | 67.5 (12-543) | 60 (28-168) | 71 (12-543) | **0.005** |
| V3 | 74 (13-472) | 50.5 (19-198) | 80 (13-472) | **0.001** |
| V4 | 66 (11-406) | 44 (19-198) | 76 (11-406) | 0.099 |
| EOS | 76.5 (16-1572) | 53 (16-121) | 85 (18-1572) | **0.044** |
| Median Troponin T (range) in ng/l | |  |  |  |
| Baseline | 11.5 (3.3-190) | 13 (3.3-37.6) | 11.4 (3.3-190) | 0.886 |
| V1 | 12.25 (3.3-116) | 16.3 (3.3-35.8) | 11.9 (3.3-116) | 0.831 |
| V3 | 11.7 (3.3-62.7) | 15.45 (3.3-62.7) | 11 (3.3-47.8) | 0.162 |
| V4 | 14.8 (3.3-64.9) | 30.55 (12.9-41.8) | 14.5 (3.3-64.9) | **0.022** |
| EOS | 11.55 (3.3-173) | 11.8 (4.2-56) | 11 (3.3-173) | 0.597 |
| Median albumin (range) in g/l | |  |  |  |
| Baseline | 38 (20-50) | 40.5 (32-48) | 37 (20-50) | **0.004** |
| V1 | 36 (18-203) | 40.5 (39-42) | 35.5 (18-203) | 0.972 |
| V3 | 38 (20-46) | 38 (34-42) | 38 (20-46) | 0.733 |
| V4 | 36 (20-43) | 35.5 (33-38) | 36 (20-43) | 0.953 |
| EOS | 37.5 (23-61) | 37 (35-38) | 37.5 (23-61) | 0.828 |

Abbreviations: CRP, c-reactive protein; HA(-), no hospital admission registered; HA(+), hospital admission registered; V1: 3-4 weeks after baseline; V3: 9-12 weeks after baseline; V4: 12-16 weeks after baseline; EOS: 6 months after baseline.

**Supplemental Table 3:** Univariate and multivariate analysis of laboratory parameters of the overall ICOG cohort.

| **Parameter** | **p-value** **Sig.** | **Hazard** **Ratio** | **95% Confidence for hazard** | | **p-value** **Sig.** | **Hazard** **Ratio** | **95% Confidence for hazard** | |
| --- | --- | --- | --- | --- | --- | --- | --- | --- |
|  |  |  | **lower value** | **higher value** |  |  | **lower value** | **higher value** |
| **Calcium V4 absolute** | 0,068 | 0,009 | 0,000 | 1,423 |  |  |  |  |
| **Magnesium V1 absolute** | 0,103 | 63125 | 0,107 | 3,71⋅10^10 |  |  |  |  |
| **CRP BL absolute** | 0,182 | 0,989 | 0,974 | 1,005 |  |  |  |  |
| **CRP V1 absolute** | 0,179 | 0,984 | 0,960 | 1,008 |  |  |  |  |
| **sIL2R BL absolute** | 0,102 | 0,998 | 0,997 | 1,000 |  |  |  |  |
| **sIL2R V4 absolute** | 0,997 | 1,090 | 0,000 | 7,35⋅10^19 |  |  |  |  |
| **Kreatinin V4 absolute** | 0,058 | 1,016 | 0,999 | 1,032 |  |  |  |  |
| **Urea BL absolute** | 0,104 | 0,856 | 0,709 | 1,033 |  |  |  |  |
| **TroponinT V3 absolute** | **0,042** | 1,048 | 1,002 | 1,096 | 0,080 | 1,049 | 0,994 | 1,107 |
| **TroponinT V4 absolute** | **0,043** | 1,065 | 1,002 | 1,131 |  |  |  |  |
| **CK V1 absolute** | 0,093 | 0,991 | 0,980 | 1,002 |  |  |  |  |
| **CK V3 absolute** | **0,021** | 0,986 | 0,975 | 0,998 |  |  |  |  |
| **CK V4 absolute** | 0,109 | 0,991 | 0,981 | 1,002 |  |  |  |  |
| **CK EOS absolute** | **0,003** | 0,974 | 0,957 | 0,991 |  |  |  |  |
| **Albumin BL absolute** | **0,035** | 1,116 | 1,008 | 1,237 |  |  |  |  |
| **Ferritin V4 absolute** | **0,016** | 1,003 | 1,001 | 1,005 |  |  |  |  |
| **Hemoglobin EOS absolute** | **0,008** | 0,714 | 0,557 | 0,917 |  |  |  |  |
| **Neutrophils V3 absolute** | **0,025** | 1,212 | 1,024 | 1,435 | 0,076 | 1,211 | 0,980 | 1,495 |
| **Neutrophils V4 absolute** | **0,004** | 1,355 | 1,100 | 1,669 |  |  |  |  |
| **Neutrophils EOS absolute** | **0,018** | 1,153 | 1,025 | 1,296 |  |  |  |  |

Abbreviations: CRP, c-reactive protein; sIL2R, soluble interleukin-2-rezeptor; CK, creatine kinase; BL, baseline; V1: 3-4 weeks after baseline; V3: 9-12 weeks after baseline; V4: 12-16 weeks after baseline; EOS: 6 months after baseline.
